# Effect of different sweeteners on the quality, fatty acid and volatile flavor compounds of braised pork

**DOI:** 10.3389/fnut.2022.961998

**Published:** 2022-08-04

**Authors:** Zhi-gui He, Ying Zhang, Ming-duo Yang, Yu-qing Zhang, Ying-ying Cui, Mi-ying Du, Dong Zhao, Hui Sun

**Affiliations:** ^1^School of Leisure and Health, Guilin Tourism University, Guilin, China; ^2^School of Tourism and Cuisine, Harbin University of Commerce, Harbin, China

**Keywords:** braised pork, fatty acid, volatile compound, *Siraitia grosvenorii*, mogroside, stevia glycoside

## Abstract

This study aimed to assess how several sweeteners (white sugar, *Siraitia grosvenorii* fruit, mogrosides, and stevia glycoside) affected the flavor, fatty acid composition, and quality of braised pork. The findings indicated that braised meat prepared with sweeteners differed from typical braised pork. When simmered for 60 min, the typical braised pork with white granulated sugar exhibited a significant cooking loss (CL) and little water content. Significantly more than in the group containing *Siraitia grosvenorii*, mogroside, and stevia glycoside, the Thiobarbituric acid (TBARS) value increased by 14.39% *(P* < 0.05). The sample in the group that included mogroside had a low CL rate. After 40 min of stewing, the lean pork has the highest L* value, but the 60-min stew sample is nicely colored and stretchy. Mogroside can prevent protein, and lipid oxidation, is thermally stable and reduces CL during stewing. Additionally, *Siraitia grosvenorii* and stevia glycosides help prevent oxidation from intensifying during stewing. When *Siraitia grosvenorii* is added, lipid oxidation is significantly inhibited, and stevia glycosides are more beneficial for enhancing meat color. With an increase in heating time, the fatty acids in braised pork reduced; the unsaturated fatty acid (UFA) of the *Siraitia grosvenorii* fruit (SF) and mg group also fell somewhat, and the UFA: SFA ratio was higher than that of the white sugar (WS) group. The SFA content of the braised meat in the stevia glycoside group was higher than that of the WS group. In all, 75 volatile flavor elements in braised pork were discovered by Gas chromatography-ion mobility spectrometry (GC-IMS). The sweetener increased alcohols, esters, and acids in the braised pork. As stewing time increased, ketones decreased, but aldehydes and esters increased. The pork formed antioxidant peptides with great nutritional value after cooking. Braised pork with mogroside and stevia glycoside additions primarily have some protein color protection and antioxidant effects. This study may offer fresh perspectives on applying natural sweeteners and enhancing braised pork’s flavor.

## Introduction

Because of its vivid color, redness, distinctive flavor, alluring flavor, and texture, the braised pork is a traditional Chinese dish with a long history in Chinese cuisine (1). By selecting the proper stewing conditions and preparation techniques, such as pre-frying, it is possible to enhance the nutritional benefits of fat, particularly the color, taste, and flavor of braised pork (2). Traditional braised pork is made with pork belly, white sugar, and soy sauce; the Maillard reaction takes place during processing. This reaction will result in a distinctive flavor but will also unavoidably generate hazardous compounds, including acrylamide, heterocyclic amine, and furan (3).

Unlike starch, sugar has a higher metabolic capacity and is an excellent energy source (4). However, sufficient scientific data shows that eating a lot of hot, sugary meals can quickly result in health hazards, including tooth decay, obesity, and diabetes. People are searching for low-calorie sweeteners as sugar alternatives as they become more aware of the value of health (5). Sweeteners are compounds that can make meals sweeter. According to various sources, they can be separated into artificial and natural sweeteners (6). Consumers prefer natural sweeteners with low heat and are non-toxic compared to artificial sweeteners; this has increasingly been the research focus of the industry (7). Studies demonstrate that natural sweeteners can lower blood sugar and enhance lipid metabolism. Fruit powder, dried fruit, and fruit extract or concentrate are essential sweeteners that increase the sweetness of meals and aid in producing bioactive components (8).

Mogroside and stevia glycoside, both natural sweeteners, are typical biological resources in Guangxi. *Siraitia grosvenorii* fruit has traditionally utilized boiling water to prepare tea. In Guangxi, stewed pork or ribs prepared with *Siraitia grosvenorii* has a distinct flavor. Mogroside, the primary extract of *Siraitia grosvenorii*, has 300 times the sweetness of sucrose. It has a low temperature, high stability, and a scent of *Siraitia grosvenorii*. It has significant antioxidant action, regulates blood sugar, inhibits bacteria and inflammation, and inhibits malondialdehyde production (9). Stevia glycoside is derived from the *Stevia rebaudiana* plant, which possesses the high sweetness and low heat. It is safe for baked goods, sauces, and other items since it does not promote dental cavities. Activating glucose-induced insulin secretion β-Cell function and hypoglycemic impact can improve islet function (10). Sweeteners are frequently utilized in manufacturing industrial foods and pharmaceuticals as a replacement for sucrose, although variations in their sensory qualities have yet to be studied (11).

Previous research has shown that various plant herbs’ phenolic acids, which have potent antioxidant properties and pharmacological effects (12), can reduce the damaging Maillard reaction products. Additionally, it has been demonstrated that natural antioxidants such as tea polyphenol, flavonoid compounds, and spice extracts specifically limit the formation of hazardous chemicals during the thermal processing of food, such as baking, frying, and boiling (13). Although mogroside and stevioside may be used as natural sweeteners and are produced from medicinal and food-related plants, their antioxidant action is rarely utilized in food preparation, particularly when preparing meat products. The influence of raw material types (14), cooking techniques (15), heating temperature and time (16), condiments (17), and storage conditions (18) are now the primary topics of study on the sensory, color, flavor, and other qualities of meat products. However, no research has been conducted on how different sugar kinds affect the flavor and quality of meat products in dish preparation.

This study compares the effects of various sweeteners and stewing times on the fatty acids, volatile taste compounds, and quality of braised pork to examine the differences between them and conventional braised pork. It serves as a theoretical foundation for the deep processing technology study and application development of sugar substitute food and functional food of Siraitia grosvenorii, as well as a guide for the study of the use of natural sweeteners and antioxidants.

## Materials and methods

### Sample preparations

The pork belly in this study was obtained from Liyuan fresh food chain supermarket (Guilin, Guangxi, China). The pork belly was cut into 4 cm × 3 cm × 2 cm pieces after being washed, drained, and cooked in boiling water for 2 min. Then, the samples were stir-fried with soybean oil at 180°C for 5 min using an induction cooker (WT2202, Midea, Foshan, China). According to the basis of the weight of meat, 120% water, 4% light soy sauce, 4.2% thick soy sauce, 5.3% cooking wine, 1% salt, 2% scallion, 2% ginger, 0.4% spices, and 1.7% sweeteners (white sugar, *Siraitia grosvenorii* fruit, mogroside and stevia glycoside) were added. These mixtures were braised at 95°C, and the time was set at 40 and 60 min. The soup was collected at 150°C for 5 min until thickened. Relevant indexes such as texture, color, fatty acid and flavor compounds were detected by taking braised pork samples. The specific design of sweeteners and stewing time are shown in Table 1.

**TABLE 1:** Cooking conditions of braised pork.

Numbering	Processing methods
WS-1	Add white sugar, 40 min stewed pork
WS-2	Add white sugar, 60 min stewed pork
SF-1	Add *Siraitia grosvenorii* fruit, 40 min stewed pork
SF-2	Add *Siraitia grosvenorii* fruit, 60 min stewed pork
MG-1	Add mogroside, 40 min stewed pork
MG-2	Add mogroside, 60 min stewed pork
SG-1	Add stevia glycoside, 40 min stewed pork
SG-2	Add stevia glycoside, 60 min stewed pork

### Cooking loss

The pork was put on clean filter paper, and the soup on the surface of the meat sample was drained and weighed after 5 min of natural cooling. The cooking loss (CL) was determined as follows:


Cookinglossrate(%)=(M-1M)2/M×1100%

Where: M_1_ is the mass of raw meat, g; M_2_ is the mass of the sample after heat treatment, g.

### Moisture content

Moisture content in each group of meat samples was determined by drying to a constant weight at 105°C according to AOAC procedure 950.46 using an electric blast drying oven (9140AL; Keelrein, Shanghai, China) from the laboratory.

### Measurement of pH

One gram of braised pork was chopped, transferred to a beaker, homogenized with 10 mL of distilled water for 35 s, filtered, and tested the pH. Prior to measurement, the pH meter was calibrated using matched buffers with various values of 4.00, 6.86, and 9.18. After the pH meter reading stabilized, the pH electrode was placed into the filtrate for testing, and the result was recorded.

### Color

Using a spectrophotometer (CM-5; KonicaMinolta, Tokyo, Japan), the lightness (L*), redness (a*), and yellowness value (b*) of the fat layer and thin layer of braised pork were measured. Self-inspection, zero calibration and whiteboard calibration were conducted before use, and each group was conducted in triplicate.

### Texture profile analysis

The texture profile in terms of hardness, springiness, cohesiveness, chewiness, and gumminess of rectangular samples (2 cm length, 1 cm width, 1 cm height) cut from braised pork were assessed for texture profile analysis (TPA) using the food texture analyzer (TMS-PILOT; Food Technology Corporation, Virginia, United States). The parameters of TPA were as follows: the trigger force of 0.5 N, a test speed of 60 mm/min, a return distance of 35 mm, and deformation of 40%. The shear force is measured in single knife shear mode, and the working parameters were set: the trigger force of 2 N, a test speed of 120 mm/min and a return speed of 200 mm/min. Each group of samples is measured with three parallel samples, and the average value is taken for the calculation.

### Malondialdehyde content

The Malondialdehyde (MDA) content was determined by following the Díaz et al. (19) method with appropriate modifications. The minced meat (5 g) was homogenized in 25 mL of trichloroacetic acid and EDTA mixture (Macklint Biochemical Technology Co., Ltd, Shanghai, China) at 10,000 rpm for 30 s. Then the sample was centrifuged at the speed of 3,000 r/min for 5 min, and the supernatant was filtered with filter paper. Five milliliters of supernatant were mixed with 5 mL of 0.02 mol/L thiobarbituric acid (TBARS) solution. The reaction mixture was heated at 95°C for 30 min in a thermostat water bath cauldron, and then the sample was placed in ice for 30 min and rapidly cooled to room temperature. The absorbance was measured at 532 nm, and the TBARS value was expressed as mg MDA/kg (measured by meat sample).

### Extraction of myofibrillar protein

The extraction of myofibrillar protein (MP) was determined according to Park et al. (20), with some modifications. The minced pork meat was homogenized on ice for 60 s (divided into three times, the 20 s each time) with four volumes (w/v) of protein extract buffer (10 mmol/L Na_3_PO_4_, 0.1 mol/L NaCl, 2 mmol/L MgCl_2_, 1 mmol/L EGTA), then centrifuged at 3,500 rpm, 4°C for 15 min. The precipitate was taken out, and the crude MP was obtained by repeating twice above the operation. Then, the precipitated sample was washed three times with 4 volumes (w/v) of 0.1 mol/L NaCl under the same centrifugation conditions. Finally, the suspension was filtered through four layers of gauze and centrifuged again at 3,500 rpm, 4°C for 15 min. The final precipitate was MP, and the next test was carried out.

### Sulfhydryl content and carbonyl content

Protein oxidation, assessed on extracted MP, was evaluated using multiple indexes, including carbonyl and sulfhydryl content. The sulfhydryl content was determined according to Yang et al. (21) with minor modification. Eight milliliters of Tris-glycine solution (10.4 g/L Tris, 0.9 g/L glycine, 10 mmol/L EDTA, 8 mol/L urea, pH 8.0) was added to 1 mL diluted MP solution (5 mg/mL), then centrifuged at 4°C and 4,500 rpm for 15 min after mixing to remove insoluble protein. Then, 0.5 mL of 10 mol/L 5, 5’-dithiobis-2-nitrobenzoic acid (DTNB) was added to 4.5 mL of supernatant. The mixed samples were incubated at room temperature and away from light for 30 min, and the light absorption value was measured at 412 nm. The sulfhydryl content was calculated using the molar extinction coefficient of 13,600 L/(mol⋅cm).

Carbonyl content was measured as determined by Ortuño et al. (22). One milliliter of 10 mmol/L 2,4-dinitrophenylhydrazine (DNPH) was added to 3 mL of MP diluent (5 mg/ml). After an incubation of 1 h (shaking every 15 min), 1 mL of 20% w:w TCA solution was added. The sample was vortexed and centrifuged for 5 min at 5,000 rpm, 4°C. The supernatant was discarded, and the precipitate was washed with 1 mL of ethanol/ethyl acetate (1:1) three times to remove the unreacted reagents. Then the supernatant was dissolved in 3 mL 6 mol/L guanidine hydrochloride, shaken, and centrifuged for 3 min at 5,000 rpm, 4°C, to avoid insoluble fragments. The absorbance value of the obtained solution was measured at 370 nm, and the carbonyl content was calculated using the molar absorbance coefficient of 22,000 L/(mol ⋅ cm).

### Fatty acid analysis

The fatty acid composition of braised pork was determined using gas chromatography (GC). The lipid portion of braised pork was extracted by adding ether/petroleum (1:1, vol/vol), and the lipid extract was collected by a rotary evaporator. The extracted sample was placed in a 250 mL round-bottomed flask, and the methyl ester preparation was carried out by the boron trifluoride-methanol method according to Bazina et al. (23). Then, quantitative analysis was carried out using the standard internal technique, and qualitative analysis was carried out using the comparing retention times of mixed standards and samples of fatty acid methyl ester. The condition parameters of GC were set according to Chen et al. (24).

### GC-IMS analysis

The FlavourSpec GC-IMS flavor analysis system (G.A.S., Dortmund, Germany) is an automated headspace injection system used to analyze the volatile flavor components. The volatile flavors of braised pork cooked with various sweeteners and stewing time were tracked. Five grams of sample were weighed and placed in a 20 mL headspace bottle for analysis, and three repetitions were made for each sample. The specific system conditions are shown in Tables 2, 3. The qualitative analysis of volatile flavor substances is carried out through the NIST database and IMS database built in the software, and difference spectra and fingerprints of volatile organic compounds were constructed by Reporter and Gallery Plot programs.

**TABLE 2:** Analysis conditions.

System		Condition
FlavourSpec unit	Analysis time	30 min
	Column type	MXT-5 15 m 1 μm 0.53 mm
	Column temperature	60°C
	Carrier gas flow	N_2_
	IMS temperature IMS	45°C
The automatic headspace sampling unit	Incubation temperature	90°C
	Incubation time	15 min
	Injection volume	500 μl
	Syringe temperature	85°C
	Incubation speed	500 rpm

**TABLE 3:** System conditions of GC.

Time	E1	E2	*R*
00:00,000	150 mL/min	2 mL/min	Rec
02:00,000	150 mL/min	2 mL/min	–
10:00,000	150 mL/min	10 mL/min	–
20:00,000	150 mL/min	100 mL/min	–
30:00,000	150 mL/min	100 mL/min	Stop

### LC-MS/MS

The identification of peptides was determined using Jiang et al. (25) with few modifications. The peptide samples digested by enzymatic (trypsin, v5280) were eluted using C18 analytical columns (Acclaim PepMap RSLC, 75 μm × 15 cm, 2 μm, 100 A, nano Viper, Thermo Fisher Scientific, Palo Alto, CA, United States). The peptide solution was collected after centrifugation and dried in a vacuum.

Subsequently, the peptide is dissolved by 20 μL of solution (0.1% formic acid, 5% ANC), then centrifuged at 13,500 rpm and 4°C for 20 min after sufficient oscillation. The supernatant was transferred to the sample pipe and identified using Dionex Ultimate 3000 RSLCnano Liquid chromatography tandem mass spectrometry (LC-MS/MS) (Thermo Fisher Scientific, Palo Alto, CA, United States). The liquid phase setting parameters are as follows: mobile phase A is 0.1% formic acid, mobile phase B is 0.1% formic acid and 80% ACN. In a word, the polypeptide liquid was eluted with a linear gradient at a flow rate of 300 nL/min. The gradient elution was performed as follows: 0–5 min (5–10%B), 5–43 min (10–28%B), 43–51 min (28–38%B), 51–53 min (38–100%B), 53–60 min (100–100%B).

The separated peptides were detected online by Thermo Scientific Q Exactive PLUS (Thermo Fisher Scientific, Palo Alto, CA, United States), and the specific conditions of mass spectrometry were as follows: primary mass spectrometry parameters: resolution: 12000, AGC target: 4e5, maximum Injection time: 50 ms, scan range: 350–1550 m/z. Secondary mass spectrometry parameters: resolution: 30000, AGC target: 1e5, TopN: 20, NCE/stepped NCE: 32. In addition, carbamidomethyl (C) was specified as the fixed modification, and oxidation (M), acetyl (Protein N-term) and NEM (C) were set as variable modifications. Fragment mass error tolerance was 0.03 Da, and precursor mass error tolerance was set at 15 ppm. LC-MS/MS spectra of peptides were matched using the Swiss-Prot database against *Sus scrofa*^1^.

### Statistical analysis

The data were analyzed by one-way analysis of variance (ANOVA) and Duncan’s multiple range test, using SPSS 21.0 (IBM., Armonk, NY, United States). Results of each group were expressed as means ± standard deviations. The figures were performed by using the Origin2018 software (OriginLab, United States) and GraphPad Prism 8 (GraphPad Software Inc., United States).

## Results and discussion

### Basic indicators

The effects of the primary physical and chemical indexes of braised pork with different sweeteners in various stewing times are shown in Table 4. Concerning the types of sweeteners, the CL rate of braised pork with white sugar was significantly higher than that of other sweeteners (*p* < 0.05). From the aspect of stewing time, the CL rate increased significantly due to the extension of stewing time, which confirmed the law of the quality change in stewing pork. Namely, the meat protein was thermally deformed during heating with decreased muscle fiber density, which caused water evaporation and fat loss, eventually increasing CL (26). Besides, the higher temperature resulted in more significant CL but less MC in cooked samples (27). The MC of braised pork with white sugar decreased significantly with the extension of stewing time, and the sample added with *Siraitia grosvenorii* fruit was significantly lower than that added with mogroside and stevia glycoside at 60 min of stewing time. Previous studies have indicated that mogroside is relatively stable and has heat and high-temperature resistance (28). Therefore, it is difficult to decompose during stewing and remains longer than other components in *Siraitia grosvenorii*. The MC of SG-2 was 43.55%, which was 6.79% lower than that of at 40 min for stewing time. The pH of WS-1 and WS-2 were 6.09 and 6.00, while the other braised pork with other sweeteners was lower than 6.00, and the pH of SF-2 was the lowest at 5.47. It indicated that the braised pork with *Siraitia grosvenorii*, mogroside, and stevia glycoside was generally acidic, which might be affected by the acidic substances such as flavonoids and saponins contained in *Siraitia grosvenorii*. Moreover, the pH of braised pork was affected by condiments such as soy sauce, cooking wine and salt during cooking (29).

**TABLE 4:** Basic physical and chemical indexes of braised pork with different sweeteners in various stewing time.

	CL	MC	pH
WS-1	23.10 ± 0.16^b^	38.85 ± 0.27^c^	6.09 ± 0.02^a^
WS-2	25.85 ± 0.55^a^	36.46 ± 0.87^d^	6.00 ± 0.01^b^
SF-1	23.15 ± 1.67^b^	38.83 ± 0.39^c^	5.78 ± 0.02^cd^
SF-2	24.69 ± 1.38^ab^	37.53 ± 0.38^cd^	5.81 ± 0.01^c^
MG-1	22.94 ± 0.72^b^	46.42 ± 0.54^a^	5.76 ± 0.06^cd^
MG-2	23.32 ± 0.34^ab^	44.90 ± 1.02^b^	5.47 ± 0.03^e^
SG-1	23.27 ± 0.51^b^	46.72 ± 0.21^a^	5.71 ± 0.01^d^
SG-2	24.11 ± 0.82^ab^	43.55 ± 0.40^b^	5.75 ± 0.02^cd^

All values are means ± SD. Significant differences between each group were indicated by different letters (p < 0.05). The following table is the same.

### Color analysis

Maillard’s reaction during cooking was affected frequently by the added sugar. The degree of Maillard reaction becomes lower when the amount of sugar added is small, which results in lighter color (30). The change of color of braised pork is shown in Table 5. The a* value of the lean layer and L* value of the fat layer of the white sugar (WS) group were significantly higher than those in other groups (*p* < 0.05). In order to achieve the effect of red and bright color, the traditional braised pork is colored and flavored through the Maillard reaction of white sugar in hot processing. The Lightness value (L*) of WS-1 and WS-2 decreased with the extension of stewing time. At the same time, the a* value of the lean layer increased, and the a* and b* values of the fat layer decreased. The drop in a* value is due to the reduction in myoglobin produced by the rise in pork’s internal temperature (31). The L* value of the fat layer is generally more significant than that of the lean layer. Dong et al. (32) manifested that the fat content significantly impacts the meat color of meat products, especially the L_*_ value, which will increase markedly with the increase of fat content. The b* value of other groups was lower than that of stewed pork with *Siraitia grosvenorii* fruit (*p* > 0.05). *Siraitia grosvenorii* contains antioxidant polyphenol, flavonoids compound and water-soluble yellow pigment, which has thermal stability and affects fat oxidation (33). However, the L* value of braised pork with *Siraitia grosvenorii* fruit decreased, and the a* value increased at stewing for 60 min. The L* of the lean layer in MG-1 was the largest and remained at 43.04 after stewing, making braised pork’s overall color brighter. The L* value of the lean layer of SG is greater than that of WS, indicating that stevia glycoside can improve the color of braised pork. Karp et al. (34) showed that the brightness, redness and yellowness of muffin skin increased by making a muffin by adding stevia glycoside, which can improve the quality of baking products.

**TABLE 5:** Color of braised pork with different sweeteners in various stewing times.

	L*		a*		b*	
	Lean layer	Fat layer	Lean layer	Fat layer	Lean layer	Fat layer
WS-1	41.67 ± 0.63^cd^	59.13 ± 0.57^a^	11.46 ± 0.82^b^	4.85 ± 0.28^ab^	20.15 ± 0.29^b^	24.79 ± 1.83^a^
WS-2	39.54 ± 0.31^de^	54.60 ± 0.87^abc^	13.76 ± 0.17^a^	4.40 ± 0.42^b^	19.71 ± 0.82^b^	23.32 ± 0.24^a^
SF-1	44.58 ± 0.29^ab^	56.09 ± 0.97^ab^	11.05 ± 0.51^bc^	5.86 ± 0.72^ab^	23.58 ± 0.83^a^	25.62 ± 1.99^a^
SF-2	37.78 ± 1.00^e^	54.74 ± 0.82^abc^	12.11 ± 0.63^ab^	6.09 ± 0.85^ab^	19.94 ± 0.63^b^	25.08 ± 1.07^a^
MG-1	46.18 ± 0.49^a^	54.75 ± 0.33^abc^	9.43 ± 0.49^c^	6.63 ± 0.49^a^	20.42 ± 0.19^b^	22.59 ± 0.28^a^
MG-2	43.04 ± 0.32^bc^	50.90 ± 1.27^c^	10.61 ± 0.95^bc^	5.41 ± 1.48^ab^	20.86 ± 1.45^b^	21.68 ± 1.73^a^
SG-1	45.22 ± 1.70^ab^	56.47 ± 0.87^ab^	10.40 ± 0.26^bc^	5.27 ± 0.31^ab^	21.33 ± 1.31^ab^	21.73 ± 1.69^a^
SG-2	43.88 ± 1.22^abc^	53.02 ± 0.79^bc^	10.35 ± 0.96^bc^	4.67 ± 0.17^ab^	21.09 ± 0.12^b^	22.71 ± 1.43^a^

### The analysis of texture

Table 6 displays the textural characteristics of braised pork using various sugars and stewing periods. The gumminess of the group added with white sugar and stevia glycoside was more excellent, while the group’s hardness, chewiness, and sheer force added with mogroside and stevia glycoside were much lower than those of WS. Due to the development of caramel by the impact of temperature and time during the process of hot processing, white sugar can boost the sweetness and consistency of meat products. This influences braised pork’s hardness, chewiness, and other textural qualities (35). The collagen in meat was heated to dissolve during the stewing procession. The cross-linking between protein molecules is reduced, resulting in the loss of MP and the fragmentation of tissue structure, which will eventually decrease the shear force of samples (36). The shear force of the lean layer of SF-2 was the lowest. It could be connected to the fact that *Siraitia grosvenorii* protease, which has a high hydrolysis activity and a stable structure, was present in *Siraitia grosvenorii* (37). Studies have shown that plant protease can improve the tenderness of meat products owing to the ability of tenderization (38). However, the effect of stewing temperature and time on the tenderness of meat might be greater in the process of stewing. Braised pork with mogroside is more malleable during the 40-min stewing stage, and the fat layer is more springy than the lean layer. Due to its antioxidant capabilities, mogroside can reduce lipid buildup, fat degeneration, and fat oxidation (39), which can significantly improve the elasticity of the fat layer in the early stage of stewing. The hardness and chewiness of the stewed pork with stevia glycoside were superior to those with white sugar, but its cohesion and springiness were marginally inferior to those with *Siraitia grosvenorii* fruit and mogroside. In other words, stevia glycoside also has the benefit of replacing sugar in enhancing the texture and quality of meat products.

**TABLE 6:** Texture quality of braised pork with different sweeteners in various stewing times.

		Hardness	Springiness	Chewiness	Cohesiveness	Gumminess	Shear force
WS-1	Lean layer	32.49 ± 3.52^a^	3.22 ± 0.39^b^	28.50 ± 0.70^ab^	0.48 ± 0.02^abc^	10.38 ± 0.08^bc^	39.28 ± 2.68^a^
	Fat layer	17.06 ± 1.02^a^	3.63 ± 0.17^b^	20.82 ± 1.67^a^	0.45 ± 0.02^cd^	10.39 ± 0.44^ab^	16.40 ± 1.64^a^
WS-2	Lean layer	19.44 ± 1.38^b^	3.52 ± 0.11^ab^	26.23 ± 0.49^b^	0.41 ± 0.02^c^	8.55 ± 0.46^cd^	25.66 ± 0.45^bcd^
	Fat layer	10.42 ± 0.58^de^	3.02 ± 0.48^bcd^	10.32 ± 0.49^c^	0.34 ± 0.03^e^	9.37 ± 0.43^bc^	10.15 ± 1.73^b^
SF-1	Lean layer	29.33 ± 0.69^a^	3.39 ± 0.10^b^	25.76 ± 0.15^b^	0.47 ± 0.02^bc^	8.18 ± 0.39^cd^	31.63 ± 5.32^abc^
	Fat layer	13.82 ± 1.44^bc^	2.81 ± 0.06^d^	16.99 ± 0.11^b^	0.52 ± 0.03^bc^	9.76 ± 0.73^ab^	14.70 ± 1.08^a^
SF-2	Lean layer	19.23 ± 1.67^b^	4.12 ± 0.08^a^	25.23 ± 0.32^ab^	0.41 ± 0.01^c^	7.45 ± 0.51^d^	21.47 ± 0.42^d^
	Fat layer	7.96 ± 0.14^e^	2.89 ± 0.04^cd^	10.27 ± 0.14^c^	0.43 ± 0.02^d^	8.76 ± 1.03^bc^	9.70 ± 0.61^b^
MG-1	Lean layer	28.95 ± 0.76^a^	3.27 ± 0.04^b^	26.68 ± 1.07^ab^	0.52 ± 0.02^ab^	10.40 ± 1.67^bc^	37.47 ± 1.79^a^
	Fat layer	12.47 ± 1.35^cd^	4.46 ± 0.14^a^	19.62 ± 1.80^ab^	0.67 ± 0.01^a^	7.83 ± 0.21^bcd^	16.93 ± 0.03^a^
MG-2	Lean layer	19.26 ± 0.32^b^	4.10 ± 0.17^a^	21.89 ± 0.85^c^	0.52 ± 0.06^ab^	10.34 ± 1.51^bc^	27.04 ± 5.28^bcd^
	Fat layer	8.43 ± 1.18^e^	3.56 ± 0.34^bc^	10.98 ± 0.58^c^	0.55 ± 0.04^b^	6.79 ± 0.51^d^	10.88 ± 0.23^b^
SG-1	Lean layer	28.44 ± 2.08^a^	3.64 ± 0.07^ab^	29.07 ± 2.21^a^	0.55 ± 0.02^a^	14.30 ± 0.53^a^	33.53 ± 1.27^ab^
	Fat layer	16.12 ± 0.78^ab^	3.00 ± 0.11^bcd^	18.87 ± 0.64^ab^	0.45 ± 0.03^cd^	10.86 ± 0.88^a^	16.12 ± 0.81^a^
SG-2	Lean layer	20.23 ± 1.86^b^	3.11 ± 0.50^b^	22.61 ± 0.07^c^	0.44 ± 0.01^c^	11.40 ± 0.56^b^	25.02 ± 0.58^cd^
	Fat layer	8.00 ± 1.60^e^	3.27 ± 0.27^bcd^	11.62 ± 1.30^c^	0.40 ± 0.05^de^	7.89 ± 0.87^bcd^	11.75 ± 0.56^b^

### Fat oxidation

**FIGURE 1:**
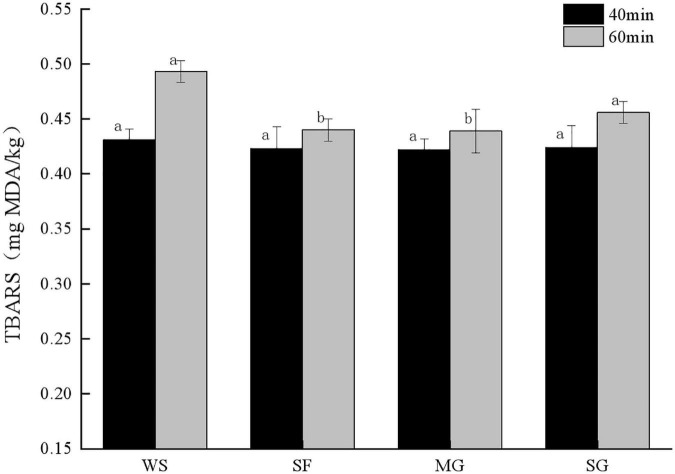
TBRAS value of stewed pork with different sweeteners in various stewing time. All values are means ± SD. Significant differences between each group were indicated by different letters (*p* < 0.05). The following figure is the same.

During heat processing, muscle protein denatured, myofibril constricted, meat moisture and quality decreased, and the TBARS value increased (40). Figure 1 demonstrates that the stewing time has a discernible effect on the TBARS value of each sample group. Specifically, the longer the cooking time, the more fat is oxidized. There was a significant difference in the TBARS value of stewed pork (*p* < 0.05) with adding different sweeteners, and the TBARS value of the WS group at stewing time for 40 min was higher than that of other groups. When the stewing time was 60 min, the TBARS value of WS increased by 14.39%, which was much higher than that of *Siraitia grosvenorii* fruit (SF), MG, and SG, indicating that this group had a significant degree of fat oxidation and produced more secondary products.

In addition, lipid content and the ratio of fatty acids impact fat oxidation (41). The TBARS concentration of samples supplemented with mogroside and stevia glycoside rose gradually. The TBARS value of samples supplemented with mogroside and stevia glycoside grew slowly because mogroside and stevia glycoside are both natural sweeteners that lower blood sugar and lipid, and their antioxidant qualities can limit fat oxidation during cooking (42). When stewed for 60 min, the MG TBARS value was 0.44 mg MDA/kg, much lower than other groups (*p* < 0.05). It demonstrates that mogroside inhibits fat oxidation and remains stable after prolonged exposure to heat. The rise in TBARS was smaller in SF than in WS and SG, which may be because polyphenols provided by *Siraitia grosvenorii* can replace free radicals and interact with unsaturated fatty acids to limit fat oxidation in meat products (43).

### Protein oxidation

**FIGURE 2:**
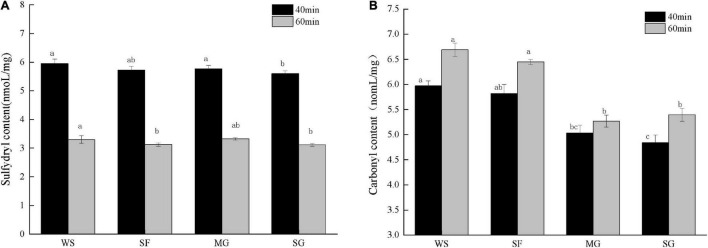
The content of sulfhydryl and carbonyl of stewed pork with different sweeteners in various stewing time. Different letters indicate significant differences (*p* < 0.05).

During cooking and processing, oxidants and tissue degradation impact meat products. In addition, meat protein is exposed to an environment including oxidative stress, resulting in the breakdown of its secondary and tertiary structures and conformational alterations (44). As the most active group in protein amino acid residues, a change in sulfhydryl concentration can represent a change in protein structure. Figure 2A depicts the effects of sulfhydryl concentration on braised pork with varying sweeteners and stewing periods. The sulfhydryl level in the 60 min stew stage was much lower than that in the 40 min stew stage due to the escalation of protein oxidation degree in the high-temperature stew process and the oxidation of free sulfhydryl to form disulfide link after heating (45). The sulfhydryl concentration of pork braised with white sugar for 40 min was more remarkable, but it fell dramatically with prolonged stewing and heating (*p* < 0.05), indicating that white sugar did not influence the loss of the sulfhydryl group during protein oxidation. However, the loss of sulfhydryl groups during stewing might be prevented by adding antioxidant sweeteners, with mogroside and stevia glycoside being the most prominent examples. Additionally, the sulfhydryl level may be connected to the amount of the four different sweeteners used. A high quantity of phenols has been found to reduce meat’s sulfhydryl content (46). The fruit of *Siraitia grosvenorii* fruit includes phenolic chemicals that affect the variation of sulfhydryl concentration.

Carbonylation of proteins is a post-translational alteration involving reactive oxygen species. Most protein oxidation happens on the side chain of amino acid residues. Oxidation can result in structural modifications to proteins, such as aromatic hydroxylation, sulfhydryl oxidation, and carbonyl formation (47). Frequently, alterations in the muscle fiber structure of meat products are the result of heat treatment. When the duration of heating is prolonged, protein denaturation increases. Generally, the greater the carbonyl concentration, the greater the protein oxidation (48). Due to the rise in protein oxidation degree caused by the lengthening of heat treatment duration, the carbonyl content rose as stewing time increased (Figure 2B).

In addition, the active carbonyl compounds initially formed by lipid oxidation and the Maillard process interact with specific amino acids on the protein side-chain via carbonyl crosslinking to make carbonyl (49). Carbonyl concentration varied significantly when stewed pork was combined with various sweeteners and cooking durations. Among them, the carbonyl content of the MG group grew less during stewing, showing that the addition of mogroside had a particular inhibitory impact on the generation of carbonyl; in other words, it may limit protein oxidation. The WS group’s carbonyl content was much greater than that of the other groups, reaching 6.69 noml/mg at the 60-min stewing stage. More reactive oxygen species will be produced during cooking, which can quickly increase carbonyl production. However, antioxidants can diminish the carbonyl concentration of cooked foods (50), which is consistent with the shift in sulfhydryl content. Cheng et al. (51) previously found that Momordica grosvenori extract (MGE) had a protective impact on the oxidative loss of pork protein, could considerably delay carbonyl formation during the storage of dried minced pork slices and decreased the loss of sulfhydryl dose-dependently. Liu et al. (52) also demonstrated that MGE exhibited significant antioxidant capacity by scavenging peroxy free radicals and intense anti-diabetic activity by inhibiting glucose-mediated protein glycosylation and cross-linking, which effectively inhibits the increase of carbonyl caused by glycosylation.

### Composition and content of fatty acid

Table 7 displays the composition and concentration of fatty acids in braised pork. Generally, the higher the unsaturated fatty acid (UFA) level of a product, the greater its nutritional value (53). The fatty acids in braised pork comprise SFA and UFA, with MUFA being the most abundant, followed by SFA, and UFA being substantially more abundant than SFA. The primary components of fatty acids in braised pork are palmitic acid (C16:0), stearic acid (C18:0), oleic acid (C18:1n9c), and linoleic acid (C18:2n6c). A similar result is reached by Da et al. (54). Cooking methods and stewing duration considerably affect the fatty acids in stewed pork. The loss of fatty acids was due to oxidation and degradation during cooking, and the SFA concentration typically declines as cooking time increases (55). Table 7 demonstrates that the SFA level of pork braised for 60 min was lower than that of pork cooked for 40 min.

**TABLE 7:** Fatty acid composition (g/100 g lipids) of braised pork with different sweeteners in various stewing times.

	WS1	WS2	SF1	SF2	MG1	MG2	SG1	SG2
C8:0	0.013 ± 0.00^a^	0.012 ± 0.00^a^	0.010 ± 0.00^a^	0.006 ± 0.00^a^	0.015 ± 0.01^a^	0.006 ± 0.00^a^	0.008 ± 0.00^a^	0.010 ± 0.00^a^
C10:0	0.048 ± 0.01^a^	0.049 ± 0.01^a^	0.050 ± 0.01^a^	0.039 ± 0.03^a^	0.046 ± 0.02^a^	0.039 ± 0.00^a^	0.043 ± 0.01^a^	0.044 ± 0.01^a^
C12:0	0.060 ± 0.01^ab^	0.065 ± 0.02^ab^	0.054 ± 0.02^b^	0.052 ± 0.01^b^	0.078 ± 0.01^a^	0.053 ± 0.01^b^	0.073 ± 0.00^a^	0.059 ± 0.01^ab^
C14:0	0.573 ± 0.04^a^	0.578 ± 0.02^a^	0.574 ± 0.03^a^	0.525 ± 0.01^ab^	0.562 ± 0.01^a^	0.525 ± 0.02^ab^	0.607 ± 0.02^a^	0.556 ± 0.03^ab^
C15:0	0.013 ± 0.01^b^	0.015 ± 0.01^b^	0.012 ± 0.00^b^	0.011 ± 0.00^b^	0.011 ± 0.00^b^	0.017 ± 0.00^b^	0.053 ± 0.01^a^	0.015 ± 0.01^b^
C16:0	9.140 ± 0.16^a^	8.150 ± 0.12^b^	9.150 ± 0.17^a^	8.500 ± 0.10^b^	9.200 ± 0.17^a^	8.640 ± 0.30^b^	9.660 ± 0.06^a^	8.940 ± 0.09^a^
C17:0	0.065 ± 0.00^ab^	0.058 ± 0.02^b^	0.065 ± 0.01^ab^	0.065 ± 0.01^ab^	0.084 ± 0.02^a^	0.070 ± 0.03^ab^	0.091 ± 0.02^a^	0.083 ± 0.02^a^
C18:0	4.520 ± 0.39^a^	3.890 ± 0.22^c^	4.210 ± 0.12^bc^	4.620 ± 0.24^a^	4.440 ± 0.20^ab^	4.350 ± 0.09^ab^	4.526 ± 0.17^a^	4.480 ± 0.19^ab^
C20:0	0.668 ± 0.04^b^	0.774 ± 0.10^a^	0.674 ± 0.04^b^	0.664 ± 0.02^b^	0.762 ± 0.07^a^	0.737 ± 0.05^a^	0.791 ± 0.01^a^	0.690 ± 0.02^ab^
C21:0	nd	nd	*Nd*	nd	nd	nd	0.986 ± 0.05^a^	0.862 ± 0.12^a^
C23:0	0.089 ± 0.01^ab^	0.091 ± 0.03^a^	0.099 ± 0.00^a^	0.110 ± 0.02^a^	0.091 ± 0.01^a^	0.118 ± 0.01^a^	0.072 ± 0.02^bc^	0.069 ± 0.01^c^
C24:0	0.006 ± 0.01^a^	nd	0.012 ± 0.00^a^	nd	nd	nd	nd	nd
SFA	15.195 ± 0.38^bcd^	13.683 ± 0.08^d^	14.910 ± 0.18^cd^	14.594 ± 0.13^cd^	15.288 ± 0.14^bc^	14.555 ± 0.35^c^	16.911 ± 0.24^a^	15.808 ± 0.44^b^
C14:1	0.110 ± 0.02^a^	0.092 ± 0.01^a^	0.074 ± 0.02^ab^	0.100 ± 0.01^a^	0.089 ± 0.01^ab^	0.096 ± 0.00^a^	0.031 ± 0.00^c^	0.020 ± 0.01^c^
C16:1	1.210 ± 0.10^a^	1.110 ± 0.18^ab^	1.190 ± 0.10^a^	1.160 ± 0.06^ab^	1.250 ± 0.05^a^	0.980 ± 0.01^b^	1.220 ± 0.04^a^	1.110 ± 0.02^ab^
C17:1	0.072 ± 0.01^ab^	0.060 ± 0.01^b^	0.080 ± 0.02^a^	0.076 ± 0.01^ab^	0.081 ± 0.01^a^	0.072 ± 0.00^ab^	0.080 ± 0.01^a^	0.070 ± 0.00^ab^
C18:1n9t	4.180 ± 0.16^ab^	4.230 ± 0.23^a^	4.280 ± 0.07^a^	3.980 ± 0.09^c^	4.390 ± 0.06^a^	4.190 ± 0.01^ab^	4.320 ± 0.03^a^	4.100 ± 0.01^ab^
C18:1n9c	13.260 ± 0.92^a^	11.260 ± 0.52^ab^	13.220 ± 0.11^a^	12.230 ± 0.20^ab^	13.660 ± 0.05^a^	12.106 ± 0.10^ab^	13.066 ± 0.06^a^	11.250 ± 0.14^ab^
C22:1n9	0.108 ± 0.01^b^	0.108 ± 0.01^b^	0.880 ± 0.03^a^	0.111 ± 0.02^b^	0.104 ± 0.01^b^	0.094 ± 0.00^b^	nd	nd
MUFA	18.941 ± 1.20^ab^	16.860 ± 0.94^c^	19.724 ± 0.25^a^	17.656 ± 0.22^bc^	19.574 ± 0.14^a^	17.538 ± 0.12^bc^	18.717 ± 0.09^ab^	16.549 ± 0.15^c^
C18:2n6c	7.700 ± 0.02^b^	5.960 ± 0.04^c^	7.890 ± 0.03^b^	7.840 ± 0.03^b^	8.200 ± 0.04^ab^	7.300 ± 0.03^b^	9.900 ± 0.05^a^	8.100 ± 0.04^ab^
C18:3n6	0.083 ± 0.01^ab^	0.086 ± 0.01^ab^	0.086 ± 0.00^ab^	0.085 ± 0.00^ab^	0.089 ± 0.00^ab^	0.087 ± 0.00^ab^	0.100 ± 0.00^a^	0.088 ± 0.00^ab^
C18:3n3	0.066 ± 0.01^a^	0.061 ± 0.00^ab^	0.070 ± 0.00^a^	0.066 ± 0.00^a^	0.071 ± 0.00^a^	0.068 ± 0.00^a^	0.070 ± 0.00^a^	0.069 ± 0.00^a^
C20:2n6	0.331 ± 0.01^b^	0.369 ± 0.02^ab^	0.369 ± 0.01^ab^	0.424 ± 0.01^a^	0.365 ± 0.01^ab^	0.349 ± 0.00^ab^	0.394 ± 0.01^a^	0.347 ± 0.00^ab^
C20:3n6	0.067 ± 0.01^ab^	0.059 ± 0.01^b^	0.070 ± 0.00^a^	0.059 ± 0.00^b^	0.069 ± 0.01^ab^	0.072 ± 0.00^a^	0.082 ± 0.00^a^	0.078 ± 0.00^a^
C20:3n3	0.027 ± 0.00^a^	nd	nd	nd	nd	nd	nd	nd
C20:4n6	0.026 ± 0.00^a^	0.011 ± 0.00^b^	0.029 ± 0.01^a^	0.021 ± 0.00^a^	0.018 ± 0.00^b^	0.016 ± 0.00^b^	0.026 ± 0.00^a^	0.012 ± 0.00^b^
C22:2n6	0.010 ± 0.00^a^	nd	0.006 ± 0.00^a^	nd	nd	nd	nd	nd
C20:5n3	0.063 ± 0.00^ab^	0.047 ± 0.01^bc^	0.098 ± 0.00^a^	0.048 ± 0.00^bc^	0.050 ± 0.00^b^	0.018 ± 0.00^c^	0.052 ± 0.00^ab^	0.060 ± 0.00^ab^
C22:6n3	0.013 ± 0.01^a^	0.012 ± 0.00^a^	0.014 ± 0.00^a^	0.012 ± 0.00^a^	0.013 ± 0.00^a^	0.020 ± 0.00^a^	0.012 ± 0.00^a^	0.012 ± 0.00^a^
PUFA	8.386 ± 0.04^d^	6.605 ± 0.05^e^	8.631 ± 0.04^c^	8.555 ± 0.03^c^	8.874 ± 0.05^a^	7.930 ± 0.04^e^	10.636 ± 0.07^a^	8.765 ± 0.04^b^
UFA	27.326 ± 1.22^bc^	23.464 ± 0.98^e^	28.355 ± 0.29^ab^	26.211 ± 0.21^cd^	28.448 ± 0.18^ab^	25.468 ± 0.15^d^	29.353 ± 0.16^a^	25.314 ± 0.17^d^
UFA:SFA	1.798 ± 0.11^ab^	1.715 ± 0.07^bc^	1.902 ± 0.02^a^	1.803 ± 0.03^ab^	1.861 ± 0.02^ab^	1.750 ± 0.04^b^	1.735 ± 0.02^bc^	1.601 ± 0.03^c^
n-3	0.169 ± 0.02^a^	0.120 ± 0.01^cd^	0.182 ± 0.00^a^	0.126 ± 0.01^bc^	0.133 ± 0.00^bc^	0.106 ± 0.00^d^	0.134 ± 0.00^bc^	0.140 ± 0.00^b^
n-6	0.516 ± 0.01^d^	0.524 ± 0.03^d^	0.559 ± 0.02^bc^	0.589 ± 0.01^ab^	0.541 ± 0.00^cd^	0.524 ± 0.01^d^	0.602 ± 0.01^a^	0.525 ± 0.01^d^
n-6/n-3	3.052 ± 0.26^e^	4.376 ± 0.35^bc^	3.066 ± 0.08^e^	4.671 ± 0.19^ab^	4.062 ± 0.10^cd^	4.940 ± 0.09^a^	4.499 ± 0.04^ab^	3.755 ± 0.02^d^

nd Indicates that no substance was detected. Different letters within a row are significantly different (p < 0.05).

Meanwhile, the SFA level of pork cooked with various sweeteners was greater than that of pork braised with white sugar (56). In addition, fatty acids in meat may reflect its nutritional worth and influence the taste development of cooked meat products. During heat processing, the Maillard reaction and fatty acid oxidation proceed rapidly, leading to the formation of several volatile chemicals and the possible reduction of fatty acids (57).

Unsaturated fatty acids can lower cholesterol and protect against atherosclerosis (58). The amount of C20:2n6 in WS and SF grew as the stewing duration increased. It has been observed that the Maillard reaction products possess the antioxidant properties of clear hydroxyl radicals and chelating metal ions, which can limit the self-oxidation of unsaturated fatty acids during processing (59). During the same stewing period, the fatty acid content of the other three groups was greater than that of the WS group for the pork braised with white sugar. The UFA of SF and MG fell somewhat, and their ratio of UFA to SFA was more significant than that of the other two groups, with SF-1 having the highest ratio at 1,902. Shen et al. (60) investigated the antioxidant capability of adding Chinese pickled and dried mustard (PDM) to steaming pork. As additional PDM increased, the ratio of unsaturated to saturated fatty acids rose, and lipid and protein oxidation reduced. The SFA concentration of pork braised in SG was greater than that of pork cooked in WS. Kaur et al. (61) shown that stevia glycoside inhibits the production of free fatty acids.

Also critical was the balance between n-3 and n-6 PUFA in the diet. N-3 PUFA has the potential to lessen the risk of cardiovascular disease, and DHA (C22:6n3) and EPA (C20:5n3) have garnered a great deal of interest due to their high nutritional value (62). The n-6/n-3 ratio of braised pork stayed between 3 and 4, and the WS was significantly less than that of other groups. The ratio of n-6 to n-3 was more excellent in WS, SF, and MG than after 40 min of stewing. Highest EPA concentration in SF1 was 0.098 g/100 g, while maximum DHA concentration in MG2 was 0.020 g/100 g. However, area, variety, and processing conditions will also impact the composition and amount of fatty acids, which distinguishes this study from others (63).

### Volatile flavor compounds

**FIGURE 3:**
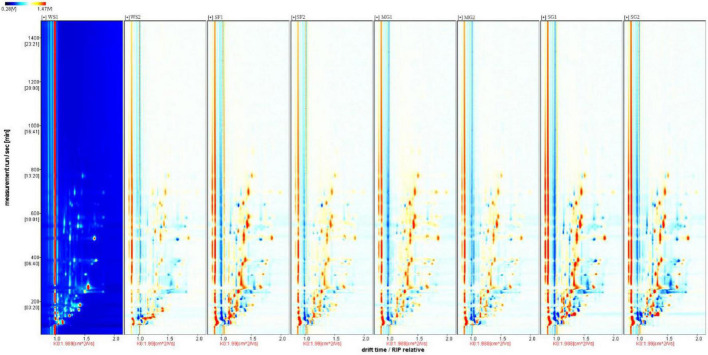
2-D GC-IMS spectra of braised pork with different sweeteners in various stewing time.

Utilizing the Gas chromatography-ion mobility spectrometry (GC-IMS) system’s high separation and sensitivity, the effects of various sweeteners and cooking time on the volatile taste components of braised pork were determined. The volatile organic chemicals in the sample can be easily recognized without needing specific sample preparation. The system recognized 75 volatile taste chemicals, including 17 aldehydes, 14 alcohols, 14 esters, 6 ketones, 2 acids, 4 hydrocarbons, and 3 nitrogen-containing compounds, based on the retention time and drift time. In Table 8, the recognized component’s name, CAS number, formula, and a few taste descriptors are included, whereas compounds with signals that could not be determined are not. Figure 3 demonstrates that the composition of volatile compounds in braised pork cooked with different sweeteners and varied stewing durations varies significantly. Using the spectrum of the braised pork sample stewed for 40 min with white sugar as a reference, a small number of red spots represent the higher compound content at the peak position, whereas blue spots represent the lower compound content at the peak position in the spectrum of the braised pork sample stewed for 60 min with white sugar. More red and blue spots emerged in the spectra of cooked pork samples sweetened with *Siraitia grosvenorii*, mogroside, or stevia glycoside. This illustrates that the stewing duration affects the volatile flavor components of the sample, but adding various sweeteners significantly affects the volatile components of braised pork. In other words, the signal strength of some chemicals is greater in samples including other sweeteners, but the concentration of specific compounds is lower in braised pork with white sugar.

**TABLE 8:** Qualitative information of characteristic flavor compounds.

Volatiles	No.	Compounds	CAS	Formula	RI	Rt	Dt	Flavor character (64)
Alcohol	1	Ethanol	C64175	C_2_H_6_O	512.1	102.0	1.051	Sweaty
	24	1-Pentanol M	C71410	C_5_H_12_O	780.9	245.9	1.250	
	25	1-Pentanol D	C71410	C_5_H_12_O	780.1	245.2	1.507	
	26	1-Pentanol T	C71410	C5H12O	780.3	245.4	1.815	
	27	2-Hexanol	C626937	C_6_H_14_O	780.3	245.4	1.282	
	28	*Cis*-2-penten-1-ol	C1576950	C_5_H_10_O	795.2	258.5	1.460	
	33	2-Hexen-1-ol M	C2305217	C_6_H_12_O	859.3	324.2	1.180	
	34	2-Hexen-1-ol D	C2305217	C_6_H_12_O	859.8	324.7	1.520	
	38	1-Hexanol	C111273	C_6_H_14_O	891.6	364.0	1.326	
	63	1-Heptanol	C111706	C_7_H_16_O	1001.8	543.1	1.389	
	65	Octan-2-ol	C123966	C_8_H_18_O	1002.8	545.1	1.435	
	69	Octen-3-ol	C3391864	C_8_H_16_O	1010.2	560.1	1.161	Mushrooms
	76	Benzylalcohol	C100516	C_7_H_8_O	1039.4	623.4	1.325	
	78	1,8-Cineole M	C470826	C_10_H_18_O	1045.2	637.0	1.289	Green
	79	1,8-Cineole D	C470826	C_10_H_18_O	1046.6	640.2	1.731	Green
	86	2-Ethyl-1-hexanol	C104767	C_8_H_18_O	1058.0	667.8	1.420	
	91	1-Octanol	C111875	C_8_H_18_O	1070.3	698.9	1.458	
	97	1-Non-anol	C143088	C_9_H_20_O	1163.9	988.8	1.535	
Ketones	5	2,3-Butanedione	C431038	C_4_H_6_O_2_	592.9	131.0	1.164	
	6	2-Butanone	C78933	C_4_H_8_O	600.9	134.4	1.252	Spicy
	13	1-Penten-3-one	C1629589	C_5_H_8_O	656.3	161.0	1.316	
	18	Acetoin M	C513860	C_4_H_8_O_2_	735.0	209.8	1.205	
	19	Acetoin D	C513860	C_4_H_8_O_2_	734.6	209.5	1.330	
	41	2-Heptanone M	C110430	C_7_H_14_O	899.3	374.1	1.259	Fruity
	42	2-Heptanone D	C110430	C_7_H_14_O	900.1	375.3	1.630	Fruity
	61	1-Octen-3-one M	C4312996	C_8_H_14_O	995.1	529.9	1.266	
	62	1-Octen-3-one D	C4312996	C_8_H_14_O	995.4	530.5	1.684	
Aldehyde	7	Butanal	C123728	C_4_H_8_O	607.8	137.4	1.292	Green fruity
	11	3-Methylbutanal M	C590863	C_5_H_10_O	660.7	163.4	1.407	Malty
	12	3-Methylbutanal D	C590863	C_5_H_10_O	656.3	161.0	1.316	Malty
	15	Pentanal M	C110623	C_5_H_10_O	695.2	183.3	1.180	Spicy, fruity
	16	Pentanal D	C110623	C_5_H_10_O	696.7	184.3	1.423	Spicy, fruity
	22	2-Pentenal(E) M	C1576870	C_5_H_8_O	757.9	227.1	1.110	
	23	2-Pentenal(E) D	C1576870	C_5_H_8_O	756.2	225.7	1.361	
	30	Hexanal M	C66251	C_6_H_12_O	799.8	262.7	1.254	Grassy
	31	Hexanal D	C66251	C_6_H_12_O	801.3	264.0	1.342	Grassy
	32	Hexanal T	C66251	C_6_H_12_O	800.6	263.4	1.563	
	43	4-Heptenal(Z)	C6728310	C_7_H_12_O	904.4	381.1	1.150	
	44	Methional	C3268493	C_4_H_8_OS	909.0	387.4	1.403	
	45	Heptanal M	C111717	C_7_H_14_O	909.8	388.6	1.328	Fruity
	46	Heptanal D	C111717	C_7_H_14_O	909.8	388.6	1.695	Fruity
	52	2-Heptenal (E) M	C18829555	C_7_H_12_O	972.9	488.6	1.257	
	53	2-Heptenal(E) D	C18829555	C_7_H_12_O	972.9	488.6	1.671	
	57	Benzaldehyde M	C100527	C_7_H_6_O	990.1	520.2	1.150	Fruity, berry
	58	Benzaldehyde D	C100527	C_7_H_6_O	989.7	519.4	1.468	Fruity, berry
	71	Octanal M	C124130	C_8_H_16_O	1021.8	584.5	1.400	Fatty, soapy
	72	Octanal D	C124130	C_8_H_16_O	1022.1	585.1	1.825	Fatty, soapy
	75	2,4-Heptadienal (E,E)	C4313035	C_7_H_10_O	1029.8	601.9	1.188	
	81	Phenylacetaldehyde	C122781	C_8_H_8_O	1048.9	645.9	1.257	
	88	2-Octenal(E) M	C2548870	C_8_H_14_O	1069.6	697.3	1.334	
	89	2-Octenal(E) D	C2548870	C_8_H_14_O	1069.3	696.3	1.820	
	92	*n*-Nonanal M	C124196	C_9_H_18_O	1097.6	773.4	1.469	Greasy, orange
	93	*n*-Nonanal D	C124196	C_9_H_18_O	1098.0	774.6	1.511	Greasy, orange
	94	*n*-Nonanal T	C124196	C_9_H_18_O	1097.6	773.4	1.942	
	95	[*E*]-2-nonenal	C18829566	C_9_H_16_O	1133.6	883.9	1.409	
	99	2,4-decadienal	C2363884	C_10_H_16_O	1249.4	1357.8	1.413	
Esters	8	Ethylacetate M	C141786	C_4_H_8_O_2_	623.0	144.4	1.099	Pineapple
	9	Ethylacetate D	C141786	C_4_H_8_O_2_	621.0	143.4	1.339	Pineapple
	17	*n*-Propylacetate	C109604	C_5_H_10_O_2_	734.5	209.5	1.160	Pear, strawberry
	20	Ethylbutanoate M	C105544	C_6_H_12_O_2_	756.9	226.3	1.219	Pineapple
	21	Ethylbutanoate D	C105544	C_6_H_12_O_2_	756.9	226.3	1.555	Pineapple
	29	Ethyl2-methylbutanoate	C7452791	C_7_H_14_O_2_	799.1	262.0	1.658	
	35	Isobutylpropionate M	C540421	C_7_H_14_O_2_	859.9	324.9	1.296	
	36	Isobutylpropionate D	C540421	C_7_H_14_O_2_	858.9	323.7	1.703	
	39	Isoamyl acetate	C123922	C_7_H_14_O_2_	896.4	370.2	1.753	Apple, pear and banana
	50	Pentyl acetate	C628637	C_7_H_14_O_2_	941.1	435.2	1.316	
	64	Ethyl hexanoate	C123660	C_8_H_16_O_2_	1001.9	543.1	1.794	Pineapple
	77	γ-Hexalactone	C695067	C_6_H_10_O_2_	1040.1	625.2	1.190	
	80	Isoamyl butyrate	C106274	C_9_H_18_O_2_	1045.9	638.7	1.398	
	82	Butyl2-methylbutanoate	C15706737	C_9_H_18_O_2_	1049.5	647.1	1.372	Pear, pineapple
	87	Diethylmalonate	C105533	C_7_H_12_O_4_	1068.7	694.7	1.249	
	90	Hexyl propanoate	C2445763	C_9_H_18_O_2_	1069.5	696.9	1.990	Fruity
	98	Hexyl butanoate	C2639636	C_10_H_2_0O_2_	1205.7	1154.7	1.488	
Acids	49	2-Methylbutyric acid	C116530	C_5_H_10_O_2_	913.3	393.5	1.215	
	59	3-Methylpentanoic acid	C105431	C_6_H_12_O_2_	990.2	520.3	1.282	
Hydrocarbons and their derivatives	56	δ-3-Carene	C13466789	C_10_H_16_	986.7	513.8	1.215	
	66	*p*-Cymene	C99876	C_10_H_14_	1004.8	549.0	1.730	Mild, pleasant
	67	*n*-Butylcyclohexane	C1678939	C_10_H_20_	1008.2	555.8	1.258	
	85	2-Methoxy-phenol	C90051	C_7_H_8_O_2_	1054.6	659.4	1.246	
Nitrogenous compounds	3	Ammonia M	C7664417	H_3_N	561.4	118.6	0.846	
	4	Ammonia D	C7664417	H_3_N	566.7	120.6	0.891	
	60	2,4,6-trimethyl-pyridine	C108758	C_8_H_11_N	994.5	528.7	1.585	
	70	2-Ethyl-5-methylpyrazine	C13360640	C_7_H_10_N_2_	1019.2	578.8	1.666	Whisky

**TABLE 9:** Antioxidant site of peptides derived from proteins after trypsin hydrolyzed.

Protein	Annotated sequence	Position	BIOSEP sequences	Activity	Quality PEP
ATP-dependent 6-phosphofructokinase	ALVFQPVTELK	A0A286ZIJ9[747–757]	LK[10–11]	Antioxidation	0.00026007
ATP synthase subunit	VELVPPTPAEIPTAIQSLK	A0A5G2QK91[31–49]	EL[2–3], LK[18–19]	Antioxidation	7.3605E-05
Alpha-1,4 glucan phosphorylase	APNDFNLK	A0A286ZMZ9[247–254]	LK[7–8]	Antioxidation	0.014763
Peroxiredoxin-2	ASGNAHIGKPAPEFQA TAVVNGAFK	A0A287AJ76[2–26]	KP[9–10]	Antioxidation	6.4107E-13
Phosphoglycerate mutase	FCSWVDQK	A0A286ZQ31[22–29]	FC[1–2]	Antioxidation	0.041327
	FCGWFDAELSEK	B5KJG2[22–33]	FC [22–23]	Antioxidation	3.0511E-06
	MEFDICYTSVLK	B5KJG2[50–61]	SVL[58–60]	Antioxidation	9.1214E-05
Ig-like domain-containing protein	DNSQNTAYLQMNSLR	A0A287BAB3[73–87]	AY[7–8]	Antioxidation	5.1175E-13
	ADAKPSVFIFPPSK	F1STC5[107–120]	KP[38–39]	Antioxidation	0.0039453
Myosin light chain 1	KPAAAAAPAPAPAPAP APAPAPPKEEK	A0A287BJF1[9–35]	KP[1–2]	Antioxidation	3.3804E-41
Multifunctional fusion protein	AGKPVICATQMLESMIK	A0A287AQJ5[926–942]	KP[3–4]	Antioxidation	2.165E-17
	ADYNVLPASENPLLR	F1SHX0[354–368]	LLR[13–15]	Antioxidation	2.5815E-07
Myosin binding protein C1	VIYQGVNTPGQPVFLEGQQQL	A0A287B5J2[1213–1233]	IY[2–3]	Antioxidation	6.942E-05
	EWSVGEPPAGEEQDKQNA NSQLSTLFVEKPQSGEVK	A0A5G2QM90[39–74]	KP[29–30]	Antioxidation	0.0065639
Malate dehydrogenase, mitochondrial	AGAGSATLSMAYAGAR	P00346[242–257]	AY[11–12]	Antioxidation	2.125E-15
Voltage-dependent anion-selective channel protein 3	AADFQLHTHVNDGTEFGGSIYQK	Q29380[175–197]	YQK[21–23]	Antioxidation	0.038426
Glyceraldehyde-3-phosphate dehydrogenase	VPTPDVSVVDLTCR	F1RM74[302–315]	LTC[11–13]	Antioxidation	4.5225E-16
Myosin-4	AEAHFSLIHYAGTVDYNITGWLDK	Q9TV62[576–599]	AH[3–4], YNI[16–18]	Antioxidation	1.6559E-52
L-lactate dehydrogenase A chain	ATLKDQLIHNLLK	P00339[2–14]	LK[3–4]	Antioxidation	1.1487E-07
					

**FIGURE 4:**
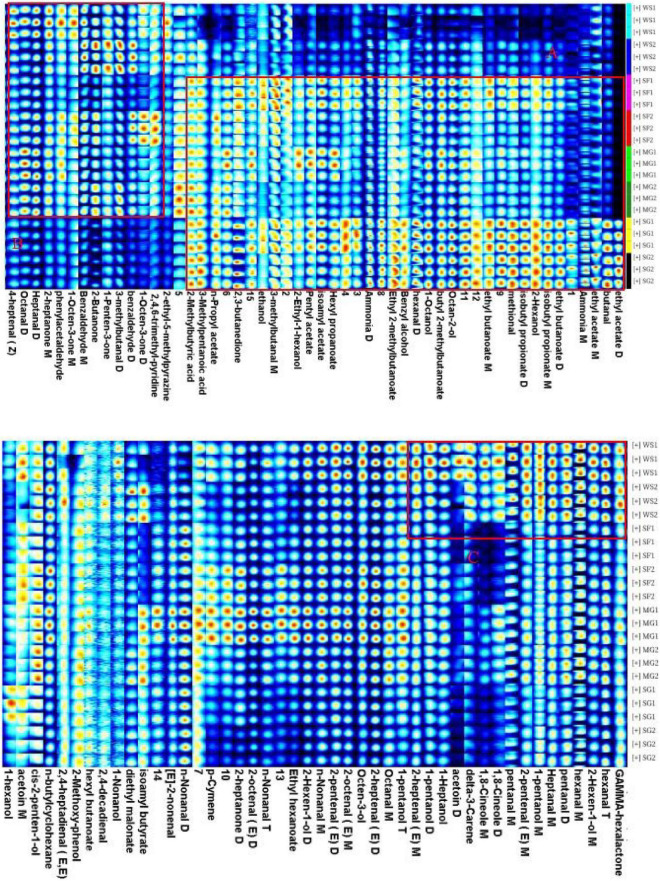
Fingerprints of characteristic flavor compounds in braised pork with different sweeteners in various stewing times generated by gallery plot.

**FIGURE 5:**
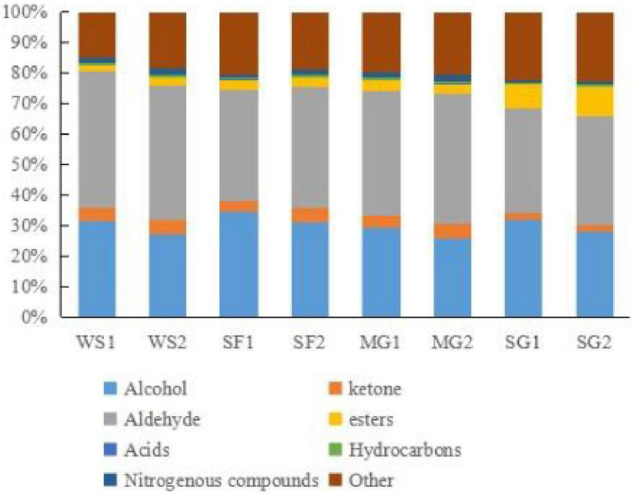
Chart of relative content of VOCs in braised pork with different sweeteners in various stewing time.

In order to examine thoroughly and intuitively the impacts of sweeteners and cooking time on the volatile substance composition of braised pork, the program may generate a fingerprint-based on the signal peak of volatile substances in each sample spectrum (Figure 4). Each row in the picture depicts the overall signal peak, whereas each column depicts the signal peak of the same volatile organic component across many samples. Each dot represents a volatile component, and the color indicates the concentration of volatile compounds. The more the content, the brighter the hue. Homologous chemicals contain monomer and dimer structures, and the concentration-dependent signals and spots are also distinct. Numbers on the fingerprint denote unidentified compounds. As shown in Figure 5, the relative concentration of volatile chemicals in each sample is also estimated based on the peak volume of volatile organic compounds. As seen in Figure 4, the signal peak intensity in regions A, B, and C is more robust in the selected region than in the other two regions.

In area A, there are esters such as butyl 2-methylbutanoate, ethyl 2-methylbutanoate, ethyl acetate, ethyl butanoate, hexyl propanoate, isoamyl acetate, isobutyl propionate, *n*-Propyl Acetate, pentyl acetate, alcohols such as 2-ethyl-1-hexanol, 2-Hexanol, 1-octanol, benzyl alcohol, ethanol, octan-2-ol, organic acids such as 2-Methylbutyric acid and 3-methylpentanoic acid, and 2,3-butanedione, methylal, butanal. Their content was lower in the braised pork with white sugar as sweetener, higher in the braised pork samples with stevia glycoside as sweetener, and more in the braised pork with *Siraitia grosvenorii* and mogroside as a sweetener and stewing time of 40 min. Aldehydes such as 3-methylbutanal, 4-heptenal (z), benzaldehyde, heptanal, octanal and phenylacetaldehyde, ketones such as 1-octen-3-one, 1-penten-3-one, 2-butanone and 2-heptanone, as well as 2-ethyl-5-methylpyrazine and 2,4,6-trimethyl-pyridine in area B are generally low in braised pork with stevia glycoside as a sweetener. The content is relatively low in the braised pork with Siraitia grosvenorii as a sweetener and stewed for 40 min. It can be seen from area C that alcohols such as 1-heptanol, 1-pentanol and 2-hexen-1-ol, aldehydes such as 2-heptenal (E), 2-pentanal (E), hexanal and pentanal, and acetoin, delta-3-carene, gamma hexalactone and 1,8-cineole are primarily in high content in braised pork with white sugar as the sweetener. Moreover, the content of 1-pentanol, *n*-nonanal, 2-heptanone, ethyl hexanoate, *p*-cymene and other substances in the braised pork added with the mogroside group was higher.

The primary volatile taste components in braised pork are aldehydes. They are generated during heat processing and derive primarily from fat breakdown. Due to their large concentration and low threshold, aldehydes have a considerable impact on the flavor of meat and contribute considerably to the scent. Pentanal has the odor of grass and banana, hexanal has a delicate odor of new grass, heptanal has the odor of fruit, and n-nonanal has the odor of grease and frying. By-products of the oxidation of oleic, linoleic, and arachidonic acids are hexanal and heptanal. Oleic acid oxidizes nonanal primarily (65).

A consequence of the breakdown of phenylalanine or linoleic acid is benzaldehyde. It quickly manifests while cooking and has a bitter almond odor. Phenylacetaldehyde is mainly metabolized via the Strecker route by phenylalanine (66). With an increase in cooking time, the degree of lipid oxidation, the generation of volatile flavor compounds, and the transformation of certain aldehydes into other taste components all rise. Figure 5 demonstrates that the amount of aldehydes in braised pork rises after 60 min of cooking; that is, both the type and amount of aldehydes increase as the cooking time increases.

Alcohols, which have a metal smell and a mushroom scent, are mainly produced through Strecker degradation and fat oxidation. In the stewing process, braised pork was rich in alcohols, with octen-3-ol, 1-pentanol, ethanol, 1-heptanol, and octan-2-ol playing a significant role in the volatile taste components. The relative alcohol concentration in samples with various sweeteners was reduced after 60 min of stewing. Contrarily, the change in the relative alcohol content of braised pork added with Siraitia grosvenorii was reasonably steady, while the relative alcohol content of braised pork added with white sugar reduced the greatest. The use of cooking wine and other ingredients may cause the stewed pork’s high ethanol concentration. Alcohols also contribute significantly to the creation of esters.

Unsaturated fatty acid breakdown, the Maillard process, and amino acid breakdown all produce ketones. They have a low threshold and can alter flavor. The primary ketones in braised pork are acetoin, 2,3-butanedione, 2, butanone, 2-butanone, and 2-heptanone. They smell like pork and cream, as is customary. They are said to improve the scent of meat items because of their distinctive qualities. 2-Butanone tastes like cream and butter, 2-Heptanone tastes like spicy blue cheese, and Acetoin have a nice flavor with a potent cream and fat aroma (67). The relative content of ketones is largely steady throughout hot processing, and there is little change when the stewing period is extended, as can be seen by the changes in fingerprint and relative content of volatile chemicals. Additionally, the varieties of braised pork with stevia glycoside and their ketones content are the lowest, and the levels of ketones in the samples from the SF2 and MG2 groups are nearly identical.

Esters are created when free fatty acids from lipid oxidation in muscle tissue mix with alcohols (68). They have a distinctive aromatic odor or a fruity scent, a high threshold, and no impact on flavor. The flavor of long-chain esters, such as hexyl butanoate, is somewhat greasy, but the flavor of short-chain fatty acid and alcohol esters, like ethyl acetate, is fruity (69). The aromas of pineapple, apple, pear, and banana may be detected in *N*-propyl acetate, ethyl butanoate, and isoamyl acetate, respectively. Esters make up a relatively tiny portion of the overall volatile flavor compounds. The braised pork and stevia glycoside had a higher ester content than the other groups, with ethyl acetate’s most excellent ester level. After stewing for 60 min, the esters in the braised pork rose due to the ongoing interaction between alcohol and acid to create esters.

Braised pork with stevia glycoside mainly comprises an acid, while pork braised with white sugar and mogroside has a higher concentration of hydrocarbons and nitrogenous substances. A few hydrocarbons are precursors to heterocyclic chemicals. The threshold of hydrocarbons in braised pork is high, and while individual hydrocarbons contribute little to flavor, the synergistic interaction of different alkanes and olefins enhances the flavor of the meat as a whole. Seasonings, including sugar, soy sauce, and cooking wine will be added during the braised pork preparation. Thermal deterioration, caramelization, and the Maillard reaction all occur during the stewing process and result in the production of pyridine, pyrazine, and sulfur compounds, which give the barbecue a mild and pleasant scent and give braised pork as it is distinctive flavor (70).

### Peptide identifications

**FIGURE 6:**
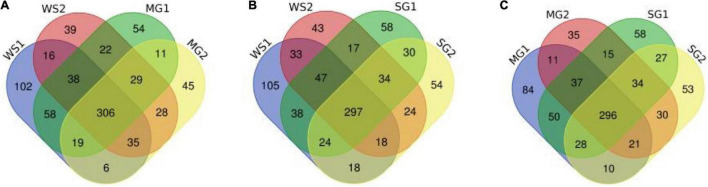
Venn image of the numbers of unique proteins from samples. **(A–C)** Respectively represent the numbers and coincidence of unique proteins between different groups of samples.

**FIGURE 7:**
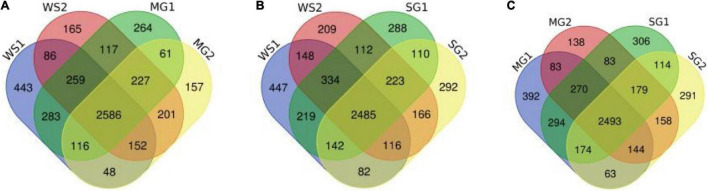
Venn image of the numbers of peptides from samples. **(A–C)** Respectively represent the numbers and coincidence of peptides between different groups of samples. Pairwise comparison highlights the differences between sample groups.

Following the enzymatic hydrolysis of braised pork, peptides were discovered using LC-MS/MS, and Figures 6, 7 display the amount of distinct proteins and peptides in each group. Seven hundred twenty-four proteins, consisting of 85% distinct proteins and 5,617 peptides, were discovered. Most of these peptides come from sarcoplasmic proteins such as phosphoglycerate kinase, L-lactate dehydrogenase A chain, alpha-1,4 glucan phosphorylase, and MPs like myosin-4, actinin alpha 2, MYL2, and desmin. Peptides are biologically active protein fragments that may supply the body with nutrients. Actin, myosin-1, myosin-4, and several glycolytic enzymes have all been demonstrated in studies to be relatively abundant proteins in pork. The amount of peptides in braised pork is more significantly impacted by the variation in stewing time than by the kind of sweetener. The kind of protein reduces as cooking time increases. It could be caused by a rise in oxidation levels during stewing, a reduction in the digestibility of animal proteins, and protein breakdown, which results in a loss of peptide segments (71). Myosin binding protein and muscle fiber structure are strongly connected. Myosin oxidation will compromise the muscle fiber structure’s integrity, lowering the meat’s quality. The elastic modulus of fibrin continues to climb, generating an elastic gel network (72). As the heating temperature rises, it will encourage the expansion of heavy myosin and the cross-linking of the myosin head and the denaturation of light myosin and the expansion of the myosin tail.

**FIGURE 8:**
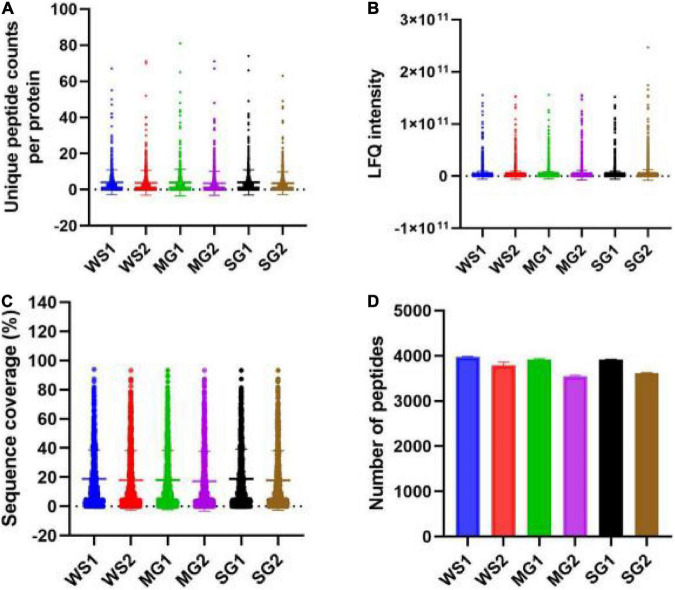
**(A)** The numbers of unique peptides counts per protein, **(B)** LFQ intensity of peptide, **(C)** sequence coverage, and **(D)** the number of peptides of samples with different sweeteners.

According to a Venn diagram, braised pork with white sugar has 2,586 peptides, braised meat with siraitin and stevia glycoside contains 2,485 peptides, and braised pork with mogroside and stevia glycoside contains 2,493 peptides (Figure 7). The amount of one peptide dropped by 180, 365, and 297 when the stewing time was increased to 60 min for the particular proteins of braised pork supplemented with white sugar, mogroside, and stevia glycoside. According to Figure 8, braised pork stewed with mogroside for 40 min had more unique peptides than other groups at the same time, while braised pork stewed with Stevia glycosides for 60 min had a higher LFQ intensity than other treatment groups and roughly the same coverage of proteome sequences. In general, increasing the number of unique peptides and proteome coverage can improve the detection of myosin complexes. Sweeteners may affect the quantity of myosin peptides in the experiment.

Bioactive peptides, such as antioxidant and antibacterial peptides, which can control physiological activity and have a variety of uses in functional foods, are typically non-toxic and very stable (73). Cooking will impact the release of bioactive peptides and the digestibility of meat products following thermal processing. When mogroside or stevia glycoside was added to braised pork, several antioxidant peptides showed more prominent expression peaks, and stew for 60 min produced higher peak intensities than stewing for 40 min. Bioactive peptides can more accurately depict the changes that braised pork undergoes throughout stewing and can serve as a foundation for evaluating the meat’s nutritional worth. The research also demonstrates a connection between glyceraldehyde-3-phosphate dehydrogenase and changes in meat color and myoglobin levels. By preventing lipid oxidation, peroxiredoxin can also lessen the impact on myoglobin oxidation (74). These proteins are primarily found in braised pork with mogroside and stevia glycoside, which may give braised meat with sugar replacements additional nutritional worth or practical benefits.

### Correlation

**FIGURE 9:**
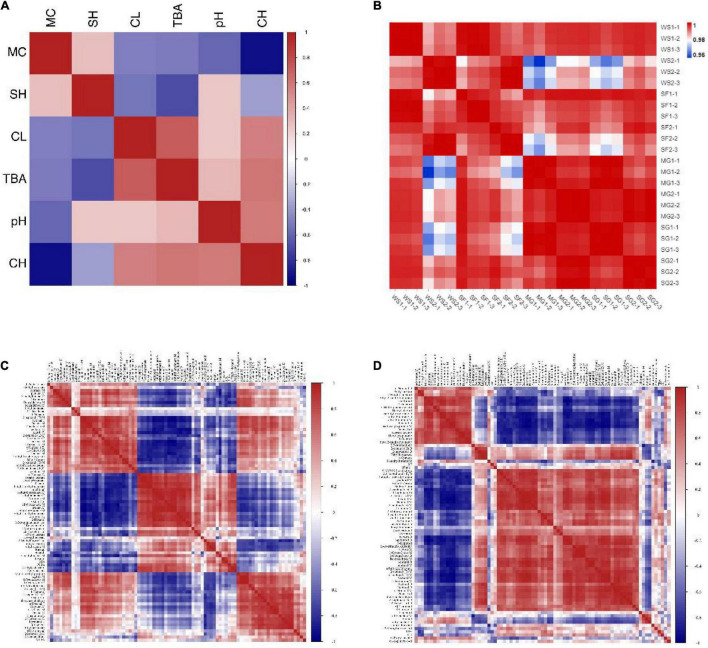
Correlation coefficients **(A)** between physical and chemical indices of braised pork, **(B)** between each group, **(C)** between various volatile components and fatty acids, and **(D)** between various volatile components and physical and chemical indices.

The physical and chemical characteristics of braised pork with various sweeteners at various stewing durations are connected (Figure 9A). Positive correlations between SH and CH, negative correlations between CH and TBA, positive correlations between CH and CL, and negative correlations between MC and CL can be found. Braised pork suffers severe CL during a protracted stewing period. The degree of oxidation increases with increasing TBA value and carbonyl concentration while decreasing sulfhydryl content. The WS2 and SF2 groups are negatively linked with other samples, and there is a positive association between other groups, according to the correlation coefficient (Figure 9B). While braised pork does vary in quality during the first 40 min of stewing, the difference becomes more noticeable after the first 60 min.

Figure 9C, which depicts the results of a correlation analysis between volatile substances, fatty acid changes, and physical and chemical indexes, revealed that PUFA was positively correlated with octan-2-ol, *n*-nonanal, butanal, and 1-hexanol, negatively correlated with 2-butanone, 1,8-cineole, and acetin, MUFA was positively correlated with 2-octanal (E), hept Aldehydes, ketones, and other chemicals all depend on fatty acids as their precursors. UFA has a more significant effect on taste compounds than SFA does. Due to the oxidation and breakdown of fat during the stewing process, aldehydes, alcohols, and ketones are more likely to be formed. Among them, aldehydes are the primary byproducts of the breakdown of unsaturated fatty acids. The relative abundances of butyric acid and nonanoic acid were positively influenced by MUFA and C18:1n9c, according to prior research.

However, there was a negative correlation between PUFA and C18:2n6c and the relative amounts of butyric acid, (E) – 2-octanoic acid, capric acid, and acetic acid (75). Some alcohols and acids continue to react to generate esters during a prolonged stewing process, which causes a drop in alcohols and a rise in esters. While TBA and carbonyl concentration were favorably connected with volatile flavoring agents like aldehydes and alcohols, SH showed a negative correlation with dimethyl malonate and isoamyl butyrate (Figure 9D). Fatty acids, TBA, sulfur concentration, and other indicators correlate strongly with volatile chemicals. They will react during the braised pork’s stewing process and particularly affect its quality.

## Discussion

Pork braised with white sugar has a high CL rate, low MC, and a pH significantly higher than other sweeteners. The L* value of this set of samples was reduced as the stewing duration rose, the a* value of the meat’s lean layer increased, the a* and b* values of the fat layer declined, and the color of the meat was bright red with different spring and a chewiness. According to Mena et al. (76), the texture of cooked pork products is influenced by pH, cooking techniques, and food additives. While cooking causes some components in meat to be lost, it may also enhance flavor and taste. The sample that had mogroside added had a low CL rate and a pH under 6.00, and the lean meat layer’s L* value was at its maximum after 40 min of stewing. After 60 min of stewing, the sample’s overall color was brilliant, the springiness was good, and the level of softness was moderate, suggesting that mogroside might be used in place of white sugar to make braised pork with better sensory attributes.

Due to the effects of heating temperature and duration during stewing, lipid and protein oxidation in pork happens. The amount of oxidation increased with longer heating times, increasing TBARS value, carbonyl content, and decreasing sulfhydryl content. The amount of fatty acids, particularly SFA, is also reduced with longer heating times. The carbonyl content was the lowest at 60 min, and the changes in TBARS value, SH, and CH of stewed pork with mogroside were noticeably less than those of other groups. Mogroside possesses strong thermal stability and can prevent oxidation during stewing, as seen from the graph. The UFA: SFA ratio was similarly higher than that of braised pork with other sweets, suggesting that natural antioxidants have a protective impact on fatty acids. The unsaturated fatty acids of SF and MG were significantly reduced, and the UFA: SFA ratio was also higher. According to research, fruit extract contains natural antioxidant active ingredients that can block fat oxidation, help preserve meat, and maintain the fatty acid composition of meat products in a stable state (77).

Additionally, stevia glycoside was utilized as a natural sweetener and antioxidant. The samples produced by varied stewing periods are superior to those containing white granulated sugar in terms of hardness, chewiness, and other characteristics. However, their springiness and cohesiveness were somewhat lower than those of the SF and MG groups, demonstrating that stevia glycoside has the same texture and quality advantages as a sugar replacement. Historically, stevia was primarily employed in dessert creation and less frequently in meat preparation. During various phases of stewing, the L* value of the lean layer in this group was more significant than that of the group using white granulated sugar, showing that stevia glycoside can improve meat sheen and color. The TBARS value and carbonyl content of stewed pork with stevia glycoside were much lower than those of white granulated sugar. The change of SH and the other change trend were likewise more diminutive than white granulated sugar, showing that stevia glycoside has antioxidant characteristics and may suppress the oxidation reaction during the cooking process.

As a fruit sweetener, *Siraitia grosvenorii* may enhance the flavor and quality of stewed pork, and the CL rate and water content of braised pork are more than in the white sugar group. When adding Siraitia grosvenorii to pork stew for 60 min, the sheer force attained its minimal quality and texture level. Due to the presence of water-soluble pigment in Siraitia grosvenorii, the b* value is much greater than that of other groups. After 60 min of stewing, the pork stewed with Siraitia grosvenorii had a low TBARS value, and its sulfhydryl and carbonyl content was lower than that of the white granulated sugar group and more than that of the other two groups. Siraitia grosvenorii includes other components, such as polyphenols and flavonoid compounds (78), which can significantly decrease lipid oxidation and protein oxidation during stewing.

There are considerable changes in the taste ingredient composition of pork cooked with various sugars. Under the impact of stewing duration, the composition and concentration of flavoring ingredients alter proportionally. Aldehydes, alcohols, and ketones are the primary volatile taste components of braised pork after cooking. Increase the pork stew’s alcohols, esters, and acids using sweeteners. Aldehydes and esters rose when the stewing time was prolonged, whereas ketones dropped. Previously, Song et al. (79) prepared braised meat with a sauce containing a Maillard reaction intermediate; the resulting flavor composition was equal to that of braised pork made with white sugar, providing a better notion for the use of sugar alternatives in braised meat. Xu et al. (80) have demonstrated that the antioxidants tert butyl hydroquinone, rosemary, and L-ascorbyl palmitat may block the degradation of optimal taste components and limit the production of odor chemicals during frying. As natural antioxidants, mogroside and stevia glycoside offer benefits as sugar substitutes; however, the oxidation level in suppressing odor formation or other phases of meat products needs more research.

Related to the protein of braised pork was its peptide. The alteration of myofibril structure significantly affects the quality of stewed meat. After braising, pork was broken down into polypeptide molecules by trypsin, which includes antioxidant peptides and has a distinct nutritional benefit. The research on polypeptide modification in meat concentrates mainly on the effects of cooking temperature, duration, and storage conditions. The sweetness was similar to that of braised pork with white sugar, which showed a declining tendency and may be more impacted by stewing duration, based only on the change in polypeptide amount. To improve the ability to recognize the unique peptides of braised meat stewed with various sweeteners, it is necessary to further study the digestive function and digestibility in the gastrointestinal tract. It is also simpler to screen the meat protein components rich in specific peptides and more nutrient-dense to more effectively realize the transformation of homemade food into industrial food.

## Conclusion

This study compared and examined the effects of various sweeteners on the quality of stewed pork at various stewing periods. The outcomes demonstrated that the quality, fatty acids, and taste of braised pork were influenced differently by sweeteners and stewing duration. The flexibility and chewability of braised pork may be diminished by prolonging the stewing period. Braised pork’s fatty acid content fell at the same time the muscle fiber structure changed, its sulfhydryl content rose, and its carbonyl content rose. By maintaining sweetness and improving the braised pig’s sensory quality, the braised pork with SF, MG, and SG can lessen oxidation and create secondary products. The mogroside-added sample has a lower level of protein oxidation, Siraitia grosvenorii fruit has a more potent inhibitory impact on lipid oxidation, and stevia glycoside has better benefits for enhancing meat color. The SF and MG group’s UFA: SFA ratio was more excellent than the WS group’s, and the SG group’s braised pork included more SFA than the WS group. By identifying peptides, one may more accurately pinpoint the peptide sequence that gives cooked meat its antioxidant benefits and improves braised pork’s nutritional value. This study can guide using sugar substitutes in meals and turning traditional cuisines into processed foods.

## Data availability statement

The supplementary data presented in this study are deposited in the Uniprot-Susscrofa repository. All original data included in this study are available upon request by contact with the corresponding author.

## Author contributions

Z-gH and YZ conducted the experiments, data analyzing, and writing—original draft preparation. Y-yC and Y-qZ contributed by the investigation and data curation. M-yD, DZ, and HS conducted the methodology, supervising data, and project administration. All authors contributed to the article and approved the submitted version.

## Footnotes

1
http://www.uniprot.org/
